# Optimal visual–haptic integration with articulated tools

**DOI:** 10.1007/s00221-017-4896-5

**Published:** 2017-02-18

**Authors:** Chie Takahashi, Simon J. Watt

**Affiliations:** 1 School of Computer Science, University of Birmingham, Birmingham, UK; 2 Wolfson Centre for Cognitive Neuroscience, School of Psychology, Bangor University, Penrallt Rd., Bangor, Gwynedd LL57 2AS UK

**Keywords:** Tool use, Multisensory integration, Optimality, Vision, Haptics, Sensory correspondence

## Abstract

When we feel and see an object, the nervous system integrates visual and haptic information optimally, exploiting the redundancy in multiple signals to estimate properties more precisely than is possible from either signal alone. We examined whether optimal integration is similarly achieved when using articulated tools. Such tools (tongs, pliers, etc) are a defining characteristic of human hand function, but complicate the classical sensory ‘correspondence problem’ underlying multisensory integration. Optimal integration requires establishing the relationship between signals acquired by different sensors (hand and eye) and, therefore, in fundamentally unrelated units. The system must also determine when signals refer to the same property of the world—seeing and feeling the same thing—and only integrate those that do. This could be achieved by comparing the pattern of current visual and haptic input to known statistics of their normal relationship. Articulated tools disrupt this relationship, however, by altering the geometrical relationship between object properties and hand posture (the haptic signal). We examined whether different tool configurations are taken into account in visual–haptic integration. We indexed integration by measuring the precision of size estimates, and compared our results to optimal predictions from a maximum-likelihood integrator. Integration was near optimal, independent of tool configuration/hand posture, provided that visual and haptic signals referred to the same object in the world. Thus, sensory correspondence was determined correctly (trial-by-trial), taking tool configuration into account. This reveals highly flexible multisensory integration underlying tool use, consistent with the brain constructing internal models of tools’ properties.

**Electronic supplementary material:**

The online version of this article (doi:10.1007/s00221-017-4896-5) contains supplementary material, which is available to authorized users.

## Introduction

The use of articulated tools is arguably a defining feature of human hand function. It is also inherently multisensory, with information available simultaneously from vision and haptics (active touch). Landmark studies have shown that when we manipulate objects directly with our hands, the senses are not used selectively, but are instead integrated statistically optimally (in the sense of producing the minimum-variance combined estimate; e.g., van Beers et al. 1998; Ernst and Banks 2002). That is, the brain takes advantage of redundancy inherent in multiple measures of the same property to improve precision beyond that possible from one signal alone (Clark and Yuille 1990; Landy et al. 1995; Ghahramani et al. 1997). Ideally, this remarkable degree of sophistication in hand function would also be achieved when we use articulated tools. However, such tools pose distinct challenges for multisensory integration by altering the relationship between the haptic sensor (hand posture) and the properties of the world. We examined whether optimal visual–haptic integration occurs in this situation, to determine whether it constitutes an underlying aspect of human tool-use expertise.

Optimal visual–haptic integration requires the brain to solve the classical sensory “correspondence problem” articulated by Molyneux and Bishop Berkeley (Berkeley 1709; see Held et al. 2011). This has two aspects. First, exploiting the redundancy in multiple signals requires knowledge of the “mapping” between estimates acquired via independent sensors (visual vs. proprioceptive stimulation), and, therefore, encoded in fundamentally unrelated units. Second, because we can feel one object while looking at another, the system must correctly determine when signals refer to the same property of the world, and only integrate those that do (or produce meaningless output). Bayesian models of multisensory integration highlight how both aspects could be solved using learned statistics of the typical mapping between signals (Ernst 2007; Körding et al. 2007; see Knill 2007, for a related approach). By knowing the way in which visual and haptic size signals, for instance, typically co-vary, one can know the likely degree to which they are redundant, and therefore, the degree to which they should be integrated (Ernst and Di Luca 2011). Moreover, similarity of pairs of signals to the typical mapping can be used to decide whether or not to integrate (Körding et al. 2007; Parise et al. 2012). Presumably, it is generally true that the similarity of estimates from two modalities is highly correlated with the likelihood that they were caused by the same property in the world. Highly discrepant visual and haptic size estimates, for example, are very unlikely to have resulted from feeling and seeing the same thing, and so constitute strong evidence that signals should not be integrated, whereas very similar estimates more likely share a common cause, and so should be integrated.

It is perhaps unsurprising if the brain knows the statistics of the normal mapping between vision and haptics (Ernst and Banks 2002), because we have had the opportunity to learn them over long, repeated exposure throughout the lifespan. Articulated tools complicate the sensory correspondence problem, however, because they alter the relationship between hand posture and object properties (at the tool tip), so disrupting the normal mapping between visual and haptic sensory signals. Even relatively simple tools, such as pliers or tongs, alter the gain between hand opening and object size, and some introduce more complex transformations, such as reversing the normal action of the hand (closing the hand causes the tool tips to open). For sensory integration to be optimal under these circumstances, the system must take account of this remapping of the haptic signal—performing what has been termed a “dramatic reinterpretation of manual hapsis” (Arbib et al. 2009)—while preserving knowledge of its statistical relationship with vision. Moreover, in tool use, discrepant hand openings and visual size signals can refer to the same object, and consistent hand openings/visual sizes can result from feeling and seeing different objects. Thus, the decision to integrate should be based on the similarity of visual and haptic sizes in the world, taking tool geometry into account.

The modal account of tool use is that tools are “incorporated” into the body schema (Head and Holmes 1911)—the postulated internal representation of the position of our body parts in space (Iriki et al. 1996; Farnè and Làdavas 2000; Maravita et al. 2003; Holmes and Spence 2004; Maravita and Iriki 2004). This account is essentially descriptive, but implies internal models that could be used to predict the movement of a tool for a given motor output, and interpret how hand posture relates to properties of the world. Although widely adopted, there is surprisingly little direct evidence for this account (Cardinali et al. 2009). For tools that merely extend the reach, grasping has been shown to change after tool use, consistent with extending the body schema (Cardinali et al. 2009), and visual–haptic integration has been shown to take account of the spatial offset between hand and tool-tips (Takahashi et al. 2009). Closer to our question, single-unit recordings from premotor cortex of a Macaque using a tool that reversed the normal grasp action suggest that such movements may be programmed in end-effector units, taking into account tool properties (Umiltà et al. 2008). However, to our knowledge, it remains unknown whether the brain can correctly determine visual–haptic correspondence across remapping of haptic signals induced by articulated tools.

Using virtual visual–haptic objects and tools, we examined: (1) whether visual–haptic integration was statistically optimal and (2) if the decision to integrate was made appropriately, across different tool geometries. Following Ernst and Banks (2002), we used improvements in size-discrimination performance when both cues were available to determine whether statistically optimal visual–haptic integration occurred. Our study probes both the flexibility of sensory integration processes per se, as well as mechanisms underlying human tool use.

## Methods

### Subjects

Seven right-handed subjects took part in the experiment (five female, two male; 19–34 years). All had normal or corrected to normal vision (including normal stereoacuity), and no known motor deficits. The study was approved by the School of Psychology Research Ethics Committee, Bangor University, and all procedures were in accordance with the Declaration of Helsinki. Subjects gave informed consent to participate, but were naïve to the precise purpose of the experiment.

### Experiment overview

The experiment consisted of two phases. In the single-modality phase, we measured the precision of each subject’s size judgements from haptics alone and vision alone. We then used these data to specify quantitative predictions for performance in the multisensory tool-use phase, where visual and haptic size information was available simultaneously, and subjects felt objects with virtual tools. Precision of the sensory estimates in all cases was determined by measuring discrimination thresholds (just-noticeable differences in size, or JNDs)—the smallest difference between two sizes that could be reliably discriminated (Ernst and Banks 2002).

This psychophysical method—employing highly intensive measurements within relatively few individuals—was important for two reasons. First, it allowed the powerful approach of making precise comparisons between observed performance and specific, quantitative predictions for the cases of optimal integration, and no integration (based on a normative model; Ernst and Banks 2002). Second, it allowed us to directly and unambiguously determine when multisensory integration occurred because, assuming that single-signal JNDs represent the best performance that can be achieved with one signal alone, any improvement with two signals can only be due to using information from both at once (i.e., exploiting the statistical redundancy in multiple signals; Ernst 2007).

### Apparatus and stimuli

**Fig. 1:**
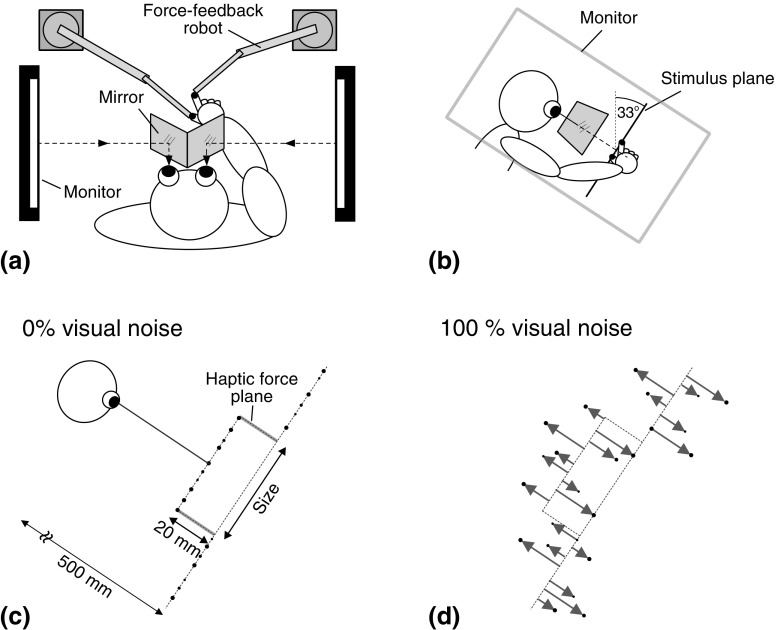
Apparatus and stimuli. **a** Plan view of the force-feedback robots and Wheatstone stereoscope components. **b** Side view of the apparatus, showing the orientation of the stereoscope and stimulus plane. **c** Side view of the visual and haptic stimuli, showing the visual object profile defined by stereoscopically presented random dots (*black circles*), and the haptic object defined by force planes (*solid lines*). The visual stimulus in this panel has 0% noise. **d** Visual stimulus with noise added. Each *dot* was displaced in depth by a random amount, drawn from a uniform distribution. Percent noise values defined the range of the sampled distribution. 100% noise, depicted here, corresponded to a range of ±100% of the depth of the bar (20 mm)

Visual size stimuli were 3-D stereoscopic images, presented via a “Wheatstone” mirror stereoscope (Fig. 1a, b), and were similar to those used by Ernst and Banks (2002). They consisted of a random-dot-defined rectangular “bar”, depicted by a raised plane 20 mm in front of a random-dot background plane. Visual size was the height of the bar, perpendicular to the line of sight (Fig. 1c). Following Ernst and Banks (2002), we manipulated the precision of visual size estimates by adding a random displacement in depth (uniform distribution) to each dot, where 100% noise denoted dot displacements drawn from a range ±100% of the separation between background and bar (Fig. 1d). A large white outline rectangle was displayed around the stimulus to aid stereoscopic fusion. The dot diameter was 4.0 mm, ± up to 1.0 mm random jitter (uniform distribution). Average dot density was 0.20 dots/mm^2^. We used anti-aliasing to achieve sub-pixel accuracy of dot positions. Because random dot placement could result in small variations from the intended size, for each stimulus, we randomly selected 3% of the dots on the raised bar and moved them to the edges. The viewing distance to the ground plane was 500 mm.

Haptic stimuli were created using two PHANToM 3.0 force-feedback robots (SenseAble Technologies, Inc.), one each for the thumb and index finger of the right hand. The haptic size stimulus consisted of two parallel rectangular force planes (stiffness = 1.05 N/mm) representing upper and lower surfaces of the rectangular bar (Fig. 1c). The 3-D positions of the tips of the digits were continuously monitored by the robots (at 1000 Hz), and touching a virtual object resulted in appropriate reaction forces, creating compelling virtual haptic surfaces. The visual and haptic “workspaces” were coincident, and the hand was not visible. Head position was stabilized using a chin-and-forehead rest.

The tools were 3D-rendered virtual pliers, comprising spheres and cylindrical rods created using OpenGL graphics primitives (see “Multisensory tool-use phase: predictions”). The tool handles followed the positions of the finger and thumb in real-time, and rotated around a pivot to open and close the tool tips. The tools translated freely in x, y, and z, but were constrained to move in the stimulus plane, because the robots sensed and produced forces in translation only. Subjects were trained to keep the “opposition space” between thumb and index finger in a similar orientation to the stimulus plane by presenting white “elastic bands” that showed the offset between the tool handle and a 1 cm sphere denoting the digit. Orienting the hand to make the elastic bands disappear resulted in the finger/thumb being in the correct orientation (trials did not commence unless they were within a tolerance of 13.5°).

Tool “gain”—defined here as the ratio of tool-tip opening to hand opening—was varied by moving the location of the pivot (see “Multisensory tool-use phase: predictions”). The tools were ~19 cm long, and different colours were used as an aid to learning/recalling the different tool geometries. We used two different tool gains: a blue tool preserved the normal 1:1 mapping between tool-tip opening and hand opening (1:1 tool) and a red 1.6:1 tool. When a tool-tip touched the haptic surface, the appropriate force was generated at the finger or thumb.

### Single-modality phase: procedure

We first measured each subject’s single-modality visual and haptic discrimination, so that we could specify precise, quantitative predictions for optimal integration, and for no integration. Following previous work, we assumed that just-noticeable differences (JNDs) in size are proportional to the standard deviation (*σ*) of the underlying size estimate in each case (Ernst and Banks 2002). Assuming the two signals are independent, with Gaussian noise, the variance of the statistically optimal integrated size estimate is given by the maximum-likelihood estimate (MLE) model of sensory integration:

1 $$\sigma _{VH}^{2}=\frac{\sigma _{V}^{2}\sigma _{H}^{2}}{\sigma _{V}^{2}+\sigma _{H}^{2}}$$

(Clark and Yuille 1990; Landy et al. 1995; Ghahramani et al. 1997; Ernst and Banks 2002). It follows from Eq. (1) that the maximum possible improvement in precision from integrating visual and haptic signals (a factor of √2) occurs when the unimodal signals have equal variance/precision. In the main multisensory experiment, we, therefore, used the data from a single-modality experiment to equate the precision of the individual signals, for each subject in each condition, so that integration effects could be assessed most clearly (Gepshtein et al. 2005; Takahashi et al. 2009).

For vision alone, we measured size-discrimination performance for 50 and 80 mm objects (the sizes used in the multisensory phase), at various visual noise levels. For haptics alone, we measured discrimination performance (without a tool) for a 50 mm object only—the size of the hand opening in our multisensory tool conditions (Fig. 3). We calculated haptic discrimination performance for an 80 mm object felt with a 1.6:1 tool simply by multiplying the discrimination threshold at 50 mm hand opening by the effects of the tool geometry. With the 1.6:1 tool, a given change in haptic object size (in the world) corresponds to a change in hand opening 1.6 times smaller than with the 1:1 tool. It necessarily follows that, for a constant hand opening (as here), sensitivity to haptic size in the world must, therefore, reduce by the same factor as the tool gain (assuming that the sensitivity to hand opening remains unchanged). For a detailed discussion of this point, and an empirical validation, see Takahashi and Watt (2014).

Size-discrimination performance was assessed using a conventional two-interval forced-choice (2-IFC) psychophysical procedure (Ernst and Banks 2002). Each trial consisted of a standard and comparison stimulus (randomly ordered), and subjects indicated which was taller. Comparison sizes were determined according to the method of constant stimuli, using eight stimulus levels. For the 50 mm (standard) object conditions, the comparison sizes were 50 mm ± increments (**Δ**sizes) of 1, 3, 6, and 9 mm, randomly ordered. For the 80 mm visual standard, we multiplied the above **Δ**sizes by 1.6 (assuming that visual size discrimination would approximately follow Weber’s law).

In the vision-only condition, the two stimulus intervals were presented for 1 s each, with a 1.6 s inter-stimulus interval. To control the haptic stimulus, which depended on the subject’s actions, each interval began with the appearance of two visual “start zones” (spheres), above and below the stimulus location, and 100 mm apart, indicating the location of the upcoming stimulus, but not its size. Placing the finger and thumb spheres in the start zones caused them to change colour, indicating that the subject should close her grasp on the stimulus. Subjects were trained to grasp the stimulus for ~1 s in each interval, and then release it, providing a near match to the vision-only presentation (Gepshtein et al. 2005; Takahashi et al. 2009). If the object was grasped for less than 900 ms, or more than 1200 ms, the message “too fast” or “too slow” appeared on the screen and that trial was discarded (with replacement). All visual information was extinguished on grasp initiation.

Subjects completed 30 repetitions of each of the eight comparison levels in both vision and haptics conditions. Thus, individual subject’s JNDs in each condition were estimated from 240 trials. Individuals differ in their sensitivity to visual noise, and so noise levels were chosen for each subject to appropriately sample their underlying function. For all subjects, we measured JNDs for at least five visual noise levels, at each of the two visual sizes (at total of least 2400 trials). No feedback was given. Subjects completed a practice session to learn the experimental procedure in both conditions, and experimental trials were completed in approximately hour-long blocks, over several days.

**Fig. 2:**
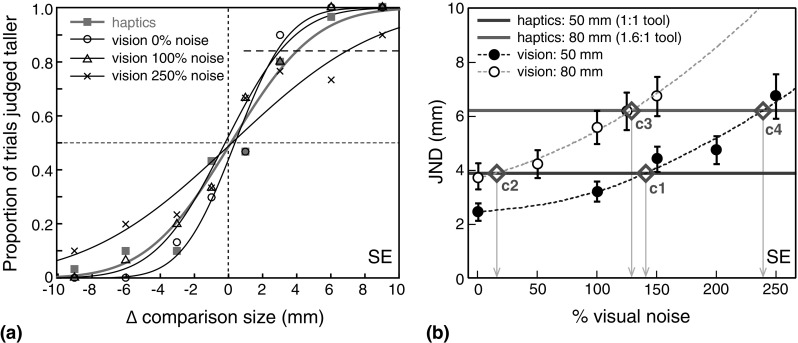
Example subject’s single-modality size-discrimination performance. **a** Example psychometric functions for haptic and visual size discrimination for the 50 mm object. The *blue symbols* denote the haptic-alone condition, and the *black symbols* denote examples of different visual noise levels for the vision-alone conditions (see legend). The data points plot the proportion of trials in which the comparison stimulus was judged as taller than the standard stimulus, as a function of the size difference between the two. The *curves* show the fitted psychometric functions. The *dashed line* in the *upper right quadrant* shows the ~84% point on the psychometric functions; the *x-axis* difference between the 50 and 84% points on the functions is equivalent to *σ* of the fitted function (i.e. the JND). **b** All measured single-cue JNDs for the same subject as **a**. The *black data points* show size JNDs for vision only, as a function of visual noise. *Closed symbols* denote 50 mm object size, and *open symbols* denote 80 mm object size. The *dashed curves* are the second-order polynomial fits to the data. *Error bars* denote ±1 standard error of the psychometric function fit. The *solid lines* show JNDs for haptics only. The *blue line* shows the measured haptic JND for a 50 mm object. The *red line* shows the calculated JND for an 80 mm object, felt with the 1.6:1 tool (see main text). Each subject’s single-cue JNDs (and associated curve-fits) were then used to determine visual noise values that matched the precision of his or her visual and haptic size estimates in each of the four conditions of the main multisensory tool-use phase. As stated in the main text, this allowed us to establish the presence or absence of optimal integration most clearly, because the maximum possible improvement in precision from integrating two signals (a factor of √2) occurs when they are equally precise (Eq. 1). The *green diamonds*, and *arrows*, denote the noise values used for this subject in each of the four conditions in the multisensory tool-use phase (labels *c1, c2*, etc refer to condition 1, condition 2, etc; see Fig. 3). (Color figure online)

Figure 2 shows an example of one subject’s single-modality discrimination performance. Figure 2a shows size-discrimination data for haptics, and from a subset of the visual noise levels tested. The curves are the best-fitting cumulative Gaussians to the psychometric data (using a maximum-likelihood criterion). Just-noticeable differences (JNDs) in size were defined as the standard deviation (*σ*) of the underlying Gaussian in each case. Figure 2b plots JNDs for all tested conditions, and the calculated haptic performance for an 80 mm object (see earlier). Variations in visual noise resulted in systematic variation in size-discrimination performance for both object sizes, allowing us to precisely match each subject’s unimodal visual precision with his or her haptic precision in the multisensory tool-use experiment.

**Fig. 3:**
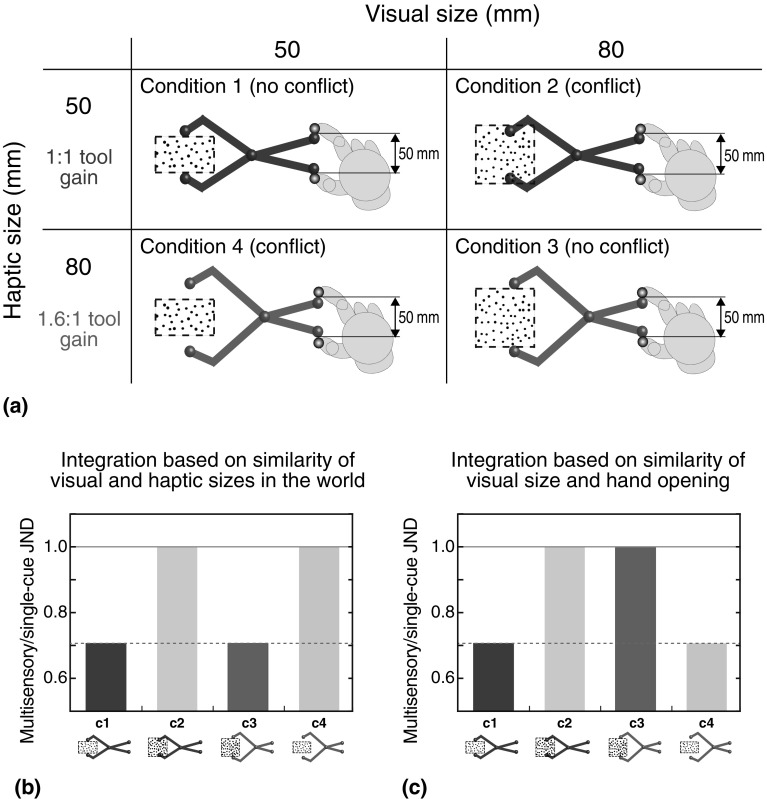
Multisensory experiment conditions, and predictions for each. **a** Cartoon of the four conditions presented. “*Conflict*” and “*no conflict*” labels refer to whether or not there was a discrepancy between visual and haptic object sizes in the world. Thus, conditions 1 and 3 were both no-conflict conditions, because visual and haptic sizes in the world were the same. Note that in condition 3, however, there was a discrepancy between visual size and hand opening. Conditions 2 and 4 were both conflict conditions, because visual and haptic sizes in the world differed. Note that in condition 4, however, visual size and hand opening were the same. The hand is shown here for illustrative purposes, but was never visible to subjects. *Grey spheres* indicated the positions of the finger and thumb. The visible tool and the *grey spheres* were extinguished before presentation of the visual and haptic size stimuli (see main text). **b** Predicted discrimination performance if the tool geometry is taken into account correctly, and visual–haptic correspondence is determined based on similarity of sizes in the world. The figure shows quantitative predictions for JNDs in each stimulus condition, expressed as a proportion of single-cue performance (i.e., normalised such that single-cue performance = 1.0). The *solid horizontal line* denotes predicted performance with no integration (i.e., performance at single-cue level), and the *dashed line* denotes predicted optimal integration performance, calculated using Eq. (1). Details of the predictions are explained in the main text. **c** Predictions for the alternative hypothesis that correspondence is determined on the basis of the similarity of visual size and hand opening (i.e., the proximal haptic signal, not taking tool geometry into account). (Color figure online)

### Multisensory tool-use phase: predictions

In the main, multisensory experiment, we measured visual–haptic size-discrimination performance while using the two different virtual tools (1:1 and 1.6:1 tool gains; Fig. 3). Each tool was used with two different visual object standard sizes: 50 and 80 mm (50 × 1.6), giving rise to four conditions, as shown in Fig. 3a.

Previously, we have shown that the brain compensates for the spatial offset between visual and haptic stimuli introduced by tools (visual object at the tool tips, and the haptic stimulus at the handles; Takahashi et al. 2009), and so our predictions are based solely on the difference in signal magnitude introduced by the changes in tool gain.

Predictions for two cases are shown in Fig. 3b, c. In both plots, the solid horizontal lines denote single-cue performance, and the dashed lines denote statistically optimal integration, computed from single-cue performance using Eq. (1). Figure 3b shows expected performance in the case of our main hypothesis that the brain takes account of tool geometry, and correctly interprets haptic size in the world, preserving the statistical properties of the signals’ correspondence. In this case, statistically optimal visual–haptic integration should occur in condition 1, where there is neither conflict between the sizes in the world, nor between hand opening and visual size (Takahashi et al. 2009). If tool geometry is taken into account, however, optimal integration should also occur in condition 3, because even though the visual size and hand opening differ, there is no conflict between visual size and haptic size in the world. Conversely, we expect no integration in condition 2, because the visual and haptic sizes are different. Thus, performance should be at the single-cue level. The hypothesis also predicts no integration in condition 4, because, again, visual and haptic sizes in the world differ, even though here the hand opening and visual size are the same. Indeed, the stimulation presented in condition 4 matches that in condition 1, with only the pre-trial exposure to the tool differing (the same is true for conditions 2 and 3).

Figure 3c presents predictions for the alternative hypothesis that integration is based on the normal mapping of visual and hand-opening signals, not taking tool geometry into account. In this case, we would expect the same pattern of results as above for conditions 1 and 2 (optimal integration and single-cue performance, respectively), because with the 1:1 tool haptic size in the world and hand opening are the same. The predictions are different for conditions 3 and 4, however. If tool geometry is not accounted for, performance would be at single-cue level (no integration) in condition 3, because the hand opening and visual size are different, and optimal integration would occur in condition 4, because the hand opening and visual size are the same.

This design, therefore, allows us to determine unambiguously whether visual–haptic integration—including the decision of whether to integrate or not—takes account appropriately of changes in visual–haptic correspondence introduced by tools. Note that both sets of predictions are based on the underlying assumption that integration is sensitive to the similarity in magnitude of visual and haptic signals per se (see “Introduction”). To our knowledge, this has not previously been confirmed empirically, and will be determined by performance in our conditions 1 and 2. In the event that the system is insensitive to signal similarity, the degree of integration will not vary with visual–haptic size conflict, and so our experiment will not be diagnostic about whether the visuo-motor system takes account of tool geometry. Assuming that the system is sensitive to visual–haptic conflict, however, conditions 3 and 4, which decouple hand opening and object size, will then indicate whether tool geometry is accounted for.

### Multisensory tool-use phase: procedure

The degree of visual–haptic integration was determined in each tool condition by measuring size-discrimination thresholds when visual and haptic signals were available simultaneously, and comparing them to the predicted performance. We again used a 2-IFC procedure, combining features of the two single-modality experiments. Each trial began with subjects seeing the tool, and inserting its tips in the “start zones” to initiate stimulus presentation (100 mm apart in the 1:1 tool conditions and 160 mm apart in the 1.6:1 tool conditions). On closing the grasp, the start zones and the visible tool were extinguished. It was important that the tool was not visible during presentation of the size stimuli, so that (1) there was not a trivial visual signal to indicate whether the tool opening matched the visual object size and (2) the precision of the visual signal was not augmented by vision of the tool, and so matched that measured in the single-modality experiment. When the force-feedback robots detected that both tool tips were in contact with the haptic object, the visual object was rendered visible. Other details were as before.

In the no-conflict conditions (visual size = haptic size; conditions 1 and 3), **Δ**size values for the comparison were the same as those used in the single-modality experiment for the 50 and 80 mm objects, respectively. For the conflict conditions, in which the visual and haptic sizes differed, **Δ**size values were determined with respect to the haptic object size. For example, for condition 2 (haptic standard = 50 mm; visual standard = 80 mm), the haptic sizes were 50 ± 1, 3, 6, and 9 mm, and the visual sizes were 80 ± 1, 3, 6, and 9 mm. **Δ**size values were the same in both modalities, so that JNDs could be computed with respect to common size units (mm). Thus, JNDs were always measured in the same units, independent of conflict or tool gain.

Within each condition, subjects again completed 30 trials per comparison size. Thus, each subject’s JND in each condition was estimated from 240 trials, and each subject completed a total of 960 trials (4 × 240) in the multisensory tool-use phase of the experiment. Again, these trials were completed in approximately hour-long blocks, spaced over several days.

Tool conditions were randomly interleaved on each trial to prevent classical adaptation effects that might otherwise result from prolonged exposure to an altered visuo-motor mapping. Prior to the main experiment, subjects completed a practice period, typically lasting several minutes, to familiarise themselves with the experiment task. After this, all subjects reported that the tools felt natural and intuitive to use.

## Results

**Fig. 4:**
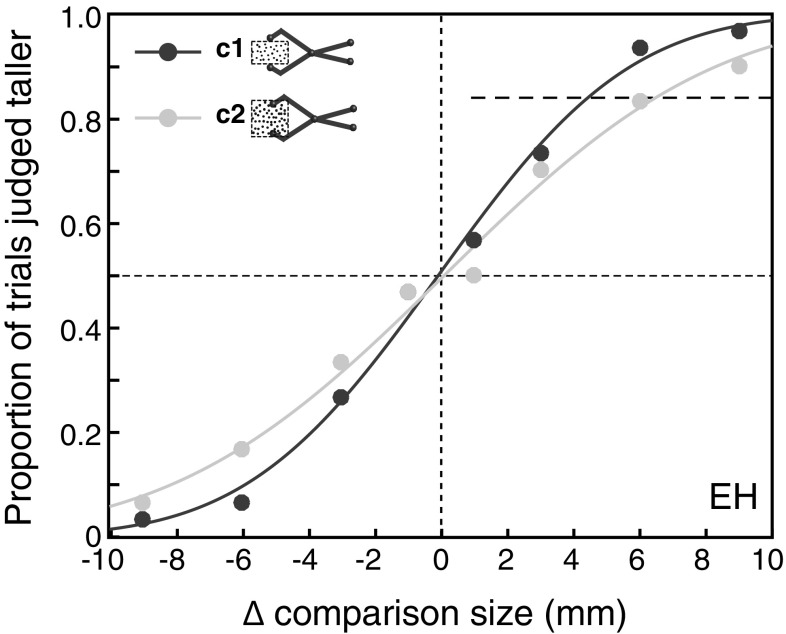
Example subject’s multisensory tool-use discrimination performance. The figure shows psychometric functions for conditions 1 and 2 (*dark*-*blue* and *light*-*blue* data points, respectively). The data points plot the proportion of trials in which the comparison stimulus was judged as taller than the standard stimulus, as a function of the size difference between the two, and the curves show the fitted psychometric functions. As shown in Fig. 2, the *dashed line* in the *upper right quadrant* shows the ~84% point on the psychometric functions, to illustrate the different JNDs (size difference between 50 and 84% point on the psychometric function). (Color figure online)

Figure 4 shows an example subject’s multisensory discrimination data for conditions 1 and 2 (1:1 tool), and the fitted psychometric functions in each case. It can be seen that this subject’s psychometric function is steeper (smaller *σ*, or JND) in condition 1 than in condition 2, indicating better size-discrimination performance when there was no visual–haptic conflict.

**Fig. 5:**
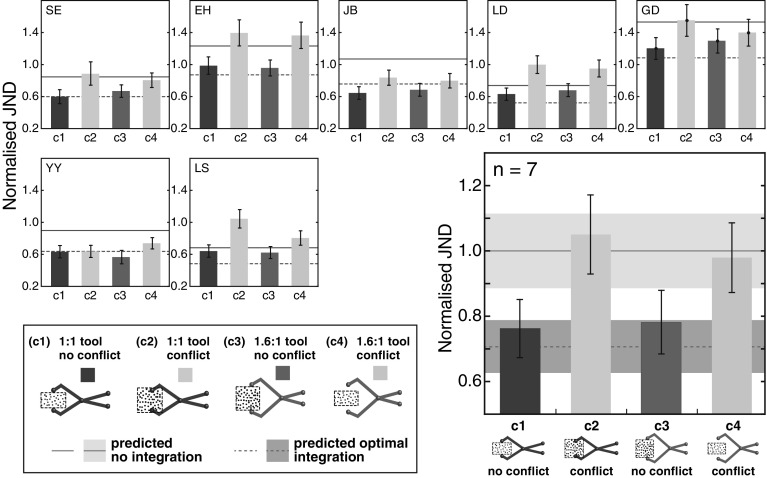
Results of the multisensory tool-use experiment. The *small panels* show individual subject’s size JNDs in each experiment condition, normalised with respect to overall average single-cue performance (see main text). The *solid black horizontal lines* denote point predictions for no visual–haptic integration. They were specified by each subject’s (matched) single-cue discrimination performance (see Fig. 2 and surrounding text). The *dashed lines* denote predicted optimal integration, calculated from the single-cue performance and Eq. (1). *Blue bars* show the 1:1 tool conditions, and the *red bars* show the 1.6:1 tool conditions. ‘*c1*’, ‘*c2*’, etc refer to the specific conditions. *Error bars* denote ±1 standard error of the estimated individual JNDs. The *larger panel* (*bottom*-*right*) shows the mean JNDs, averaged across all subjects, in the same format as the individual subject plots. The *grey zones* denote ±1 between-subject standard error of the predictions, and the *error bars* denote ±1 between-subject standard error of the observed JNDs. (Color figure online)

We determined each subject’s JND, in each condition, in the manner, as shown in Fig. 4. Figure 5 plots individual subject’s JNDs, as well as the average across all subjects. As described in “Methods”, absolute discrimination thresholds with the 1.6:1 tool (80 mm haptic object) were necessarily larger overall by a factor of 1.6 due to the effect of the tool geometry. To make it easier to make comparisons to the predictions, we normalised the data to remove this ‘baseline’ difference across the two tools. To do this, we divided each subject’s multisensory JND in each condition by the overall average (*n* = 7), matched, single-cue JND for that condition. This has the effect of scaling the average multisensory data (large panel), such that a JND of 1.0 corresponds to single-cue performance level, irrespective of condition. For the individual subject data (small panels), normalising by overall average single-cue JND (rather than within individuals) preserves inter-subject variability in absolute thresholds, including their single-cue JNDs, and optimal-integration predictions. The overall average single-cue JND for the 1:1 tool conditions (c1 and c2; 50 mm haptic object) was 4.6 mm, and so the average JND for the 1.6:1 tool conditions (c3 and c4; 80 mm haptic object) was 7.4 mm (4.6 × 1.6; see “Methods”).

In all panels in Fig. 5, predicted performance with no visual–haptic integration is denoted by a solid horizontal line (equal to the single-cue JNDs). The dashed horizontal line shows the predicted multisensory performance if the two signals were optimally integrated, computed from the single-cue performance in each condition, using Eq. (1). Note that there are no free parameters in the predictions, which could otherwise be allowed to vary to improve the fit to the data. The bars show the observed discrimination performance in the four tool conditions.

Considering the average data, there is very close, quantitative agreement between the observed JNDs and the predictions for a system that takes into account tool geometry when establishing sensory correspondence (Fig. 3b). First, there was a clear effect of visual–haptic conflict on the degree of integration across conditions 1 and 2 (1:1 tool). JNDs were near the optimal-integration prediction with no conflict (condition 1), and near single-cue levels (indicating no integration) with visual–haptic conflict (condition 2). This replicates our finding that optimal visual–haptic integration occurs in tool-use despite a spatial offset between the visual object and a haptic signal that originates at the hand/tool handles (Takahashi et al. 2009). Moreover, it demonstrates that the decision to integrate visual and haptic signals is sensitive to the similarity in magnitude of the two estimates. As noted in the predictions section, the results of conditions 3 and 4, with the 1.6:1 tool, are particularly diagnostic with respect to our hypothesis, because they decoupled hand opening and haptic object size in the world. In condition 3, there was no conflict between visual size and haptic sizes in the world, but visual size and hand opening differed (80 vs. 50 mm). Performance was near the optimal-integration prediction, indicating that visual–haptic correspondence was determined on the basis of similarity of sizes in the world. In condition 4, there was a conflict between visual and haptic sizes in the world (50 vs. 80 mm), but hand opening and visual size were the same (both 50 mm). Here, performance was at single-cue levels (i.e., visual and haptic estimates were not integrated), again consistent with correspondence being determined on the basis of visual and haptic sizes in the world.

Overall, this pattern of effects is entirely consistent with the visuo-motor system taking tool geometry into account appropriately in sensory integration. The visuo-motor system correctly determined the causal structure of the visual and haptic signals independent of tool-induced remapping of hand opening (the proximal haptic signal). That is, the system correctly determined when the signals likely referred to the same object, and so should be integrated, and when they referred to different objects, and so should not. Moreover, the finding that discrimination performance was quantitatively near optimal in the appropriate conditions (1 and 3) suggests that the information about the degree of redundancy in the two signals (their correspondence) was preserved, despite the requirement to take account of the tool geometry.

We carried out a series of planned paired *t* tests to evaluate the specific comparisons predicted by our main hypothesis. The full list is shown in Table 1, along with the predicted pattern of significant effects, assuming that tool geometry is taken into account (note that the predictions for no-integration and optimal-integration performance were not single values, but distributions made up of the individual subject’s predictions). Considering Fig. 3b, observed JNDs in no-conflict conditions should be significantly lower than the no-integration predictions, and should not differ from the optimal-integration prediction. The converse pattern should be observed for the conflict conditions. Moreover, within each tool type, JNDs should differ significantly between conflict and no-conflict conditions if visual–haptic conflict per se affected the degree of integration. Because we had directional predictions tests were one-tailed, and we used Bonferroni correction for multiple comparisons. Table 1 shows that there was complete agreement between the predicted and observed pairwise effects.

**Table 1:** Summary of expected and actual pairwise differences

Comparison	*t* Test results; i = one-tailed; Bonferroni corrected α = 0.005		
	Predicted	1:1 tool	1.6:1 tool
No-conflict vs. conflict	*	*p = 0.001*	*p = 0.001*
No-conflict vs. predicted no integration	*	*p = 0.001*	*p = 0.002*
No-conflict vs. predicted optimal integration		*p* = 0.090	*p* = 0.061
Conflict vs. predicted no integration		*p* = 0.297	*p* = 0.390
Conflict vs. predicted optimal integration	*	*p = 0.003*	*p = 0.002*

Table shows expected differences, assuming that sensory integration correctly takes account of tool geometry, and *p* values for planned *t* tests (*df* = 6) on the same comparisons

Italic values denote actual significant effects

Asterisks denote predicted significant effects

Individual subject’s data also generally show good agreement with the average pattern. Considering the predictions again (Fig. 3b), it can be seen that all the individual subject’s data to some degree resembled the predictions for taking tool geometry into account when establishing visual–haptic correspondence. Individual subject’s JNDs in the no-conflict conditions were similar to their predicted optimal-integration performance. Moreover, for five of the seven subjects (SE, EH, LD, GD, and LS), performance was close to their no-integration predictions in the conflict conditions. The remaining two (JB, and most obviously YY) showed a greater degree of integration in the conflict conditions. Results of a small-scale control experiment (see Online Resource) suggest this reflected a general increase in tolerance to visual–haptic conflict in these participants, rather than being specific to tool use/haptic remapping (also see “Discussion”). Importantly, none of the individual subject’s data resemble the predicted pattern if correspondence is determined on similarity of hand opening and visual size (Fig. 3c).

## Discussion

Our results suggest that sensory integration in tool use takes account of tool-induced remapping of haptic signals. Specifically, the finding that discrimination performance was near optimal in no-conflict conditions, and at single-signal levels (indicating no integration) for conflict trials, provides evidence that the fundamental problem of establishing sensory correspondence between visual and haptic size signals was solved correctly, independent of changes in proximal haptic signals introduced by tool geometry. That is, the brain correctly infers the causal structure of the signals, and integrates based on the similarity of haptic and visual sizes in the world, not the proximal signals.

“Compensation” for tool geometry could occur at one of (at least) two distinct levels of processing. It could be accounted for implicitly, at the level of the mapping between the two signals. That is, the unimodal signals (including the haptic estimate) remain unchanged, and only the expectation of how they relate to one another is updated. Alternatively, tool geometry could be accounted for at the level of the unimodal haptic signal, prior to integration (i.e., at the level of the mapping between the proprioceptive signals and the unimodal estimates derived from them). So far, we have considered the problem of calibrating signals from the hand and eye to each other. For the resulting integrated estimate to be meaningful, however, it must also be calibrated appropriately to the external world. That is, tool geometry must be corrected for explicitly at some stage if object size is to be estimated accurately. Moreover, unimodal haptic estimates, too, should accurately reflect the properties of the world, whether or not they are to be integrated with vision (assuming a goal of haptic perceptual constancy). Accounting for tool geometry at the level of the unimodal haptic signal therefore provides the more plausible, and parsimonious account. A single compensation process could support accurate haptic-only estimates and visual–haptic integration. In addition, it would allow visual–haptic correspondence to be determined on the basis of the same, long-established statistics of the relationship between haptic and visual sizes in the world, whether or not a tool was used.

The effect of visual–haptic conflict on sensory integration was quantitatively different for different subjects. In particular, YY showed near-optimal integration for all conditions. To explore whether these data reflected unusual “strategies” specific to our tool study, or general differences in the effect of signal conflict on integration, we ran a small-scale control experiment (see Online Resource). We measured integration as a function of visual–haptic conflict, without a tool, for subject YY, subject SE, whose data were closely matched to the predictions, and JB, who showed an intermediate pattern. In all cases, visual–haptic conflict had a continuous effect on the degree of integration, and conflict magnitude had quantitatively similar effects with and without tools. This suggests that our tool data do reflect a general operating principle of considering visual–haptic similarity, although tolerance over which integration occurs differs across subjects. YY’s pattern of results is consistent with disregarding object size, and determining correspondence solely on the basis of other properties such as temporal and spatial coincidence. Assuming the natural statistics of visual–haptic mapping, and knowledge of them, to be similar across people (an untested assumption), it is unclear why this might occur. Similar variability across subjects has been observed in the effect of spatial proximity on visual–haptic integration (Gepshtein et al. 2005; Takahashi et al. 2009). Nonetheless, for the most part, our subjects showed appropriate integration/non-integration, even though performance in our experiment would have been optimised by always integrating signals. This suggests automatic and robust use of visual–haptic similarity to determine sensory correspondence.

Our results are consistent with correctly accounting for tool geometry, even though different tools (and the presence or absence of visual–haptic conflict) were selected at random on each trial. This suggests that the visuo-motor system can “switch” very rapidly, on a trial-by-trial basis, between different hand posture/haptic size mappings (Imamizu et al. 2003; Imamizu and Kawato 2008; Botvinick et al. 2009; Beisert et al. 2010; Ingram et al. 2010). Presumably, this is achieved by learning/establishing the mappings between hand posture and object size, and then retrieving them from memory when each tool is subsequently encountered (Beisert et al. 2010). This appears distinct from “classical” adaptation, where visuo-motor mappings are gradually updated (to maintain calibration of signals that drift over time, for example). Instead, it is a situation, where a new mapping must be stored without overwriting the previous one. It is, therefore, similar to so-called “dual adaptation” (McGonigle and Flook 1978; Welch et al. 1993; Martin et al. 1996). Martin et al. (1996), for example, reported that subjects eventually learned to immediately make accurate throws to a target when switching between normal vision and wearing displacing prisms (similar to the idea that repeated exposure to one’s spectacles results in the ability to rapidly compensate for the geometrical changes induced in the retinal image when they are removed and replaced; Schot et al. 2012). We cannot determine if the acquisition of tool mappings in our study invoked the same processes as dual adaptation. Compared to dual adaptation studies, acquisition of tool mappings in our experiment seems to have occurred rapidly (Martin et al. 1996). However, we may have (largely unwittingly) created a situation that aided acquisition of multiple mappings by (1) providing online feedback of the consequences of tool geometry during movements (in the start-zones phase of each trial) (Welch et al. 1993), (2) selecting the tool type at random on each trial (Osu et al. 2004), and (3) pairing changes in contextual information with changes in visuo-motor mapping (differently coloured tools with recognisably different shapes) (Osu et al. 2004).

The ability to rapidly “switch” between tool mappings raises questions about what the cues are that specify the current mapping state (Wolpert et al. 2011). In principle, previously learned tool mappings could be identified via a range of cues, all of which were available in our experiments. For example, ‘contextual’ cues, such as the colour/appearance of the tool, could allow switching before movement even begins (Imamizu et al. 2007; van Dam and Ernst 2015a). Similarly, static spatial information about hand opening and the tool tips could allow identification. Or perhaps dynamic information about the relationship between hand and tool tips is required?

Indeed, it remains possible that mappings were not stored in our study, but entirely determined on a trial-by-trial basis (during the start-zones phase of each trial). Although our articulated tools were relatively complex compared to sticks, rakes, etc., they nonetheless had “motor equivalence” (Arbib et al. 2009): the precision grasping movements of thumb and index finger were qualitatively similar to the movement of the tool tips. This means that accounting for tool geometry by the subject required only an estimate of a mathematically straightforward gain (and spatial offset) factor. Alternatively, subjects could have learned a generic tool-gain model, and calibrated the specific relationship between hand opening and object size on-the-fly. This account—a hybrid of learning and instantaneous calibration—is appealing for at least two reasons. First, our subjects could already have acquired a generic model through normal experience with tongs, scissors etc, which could explain the rapid “acquisition” of tool mappings. Second, and more generally, learning “classes” of commonly encountered tool transformations, that can be refined rapidly to particular circumstances, simplifies the otherwise seemingly intractable problem of needing as many learned mapping states as there are tools that we use. These accounts make different predictions for the generalizability of experience with different tools, and for learning novel tool mappings (van Dam and Ernst 2015b).

Our results are consistent with haptic information being transformed, or reinterpreted, to take account of the changed relationship between hand posture and properties of the world (Arbib et al. 2009). This dynamic flexibility is interesting, because cues involving the motor system are often considered to represent a ground truth to which visual signals are calibrated. This is in part, because, unlike vision, movements must relate directly to properties of the world (captured in Bishop Berkeley’s well-known quote that “touch educates vision”; Berkeley 1709). Consistent with this, there are lots of experimental demonstrations of recalibration of visual information on the basis of touch (e.g., Ernst et al. 2000; Atkins et al. 2003; Ho et al. 2009; van Beers et al. 2011), but fewer, and generally less pronounced, examples of vision altering touch (e.g., Gori et al. 2011; Wismeijer et al. 2012). Perceiving object properties via tools creates a problem of perceptual constancy analogous to that of visual perception: hand posture is no longer matched with object properties in the normal way, and so haptic signals must be re-scaled to be accurate on their own, and appropriately cross-calibrated with visual signals. Our results suggest that when there are clear signals indicating that haptic information needs recalibrating (from seeing the tool, for example), the system readily does so.

The brain is thought to use an internal (forward) model for motor control, operating on a copy of the motor command, to predict the future position and velocity of the moving hand (Wolpert et al. 1995). If such models are extendable to include tool properties, the visuo-motor system could, in principle, predict the consequences of motor commands for movements of the tool tips (including grasp aperture). This could support efficient control of tools in the natural environment, and allow sensory correspondence to be established in our experiments on (haptic) object size in the world. There is specific evidence for the acquisition of such internal models, and for the ability to switch between them in producing motor output (e.g., Imamizu et al. 2000; Imamizu and Kawato 2008; Ogawa and Imamizu 2013). This idea is also consistent with the wider tool-use literature. It provides a potential mechanism for the phenomenological experience that we are “feeling” objects with the tips of a tool, even though the haptic signals originate at the hand, and for the specific finding that when a monkey uses a tool the receptive fields of cells responding to locations around the hand extended to include the region surrounding the tool (Iriki et al. 1996), for example. It is also generally consistent with the idea that tools are “incorporated” into the body schema (Maravita and Iriki 2004), providing a mechanism by which movements with tools could be specified in terms of the distal “goal” of controlling the end effector, independently of intermediate visuo-motor mappings (Gentilucci et al. 2004; Arbib et al. 2009), simplifying the job of movement planning and control under changing circumstances.

## Electronic supplementary material

Below is the link to the electronic supplementary material.

Supplementary material 1 (DOCX 161 KB)
