# Supplementary material for: Optimal visual–haptic integration with articulated tools
Source: Exp Brain Res. 2017 Feb 18;235(5):1361–73. doi: 10.1007/s00221-017-4896-5 (PMC5380699; doi:10.1007/s00221-017-4896-5)
Supplement: Supplementary file 1 — Supplementary material 1 (DOCX 161 KB) [file 221_2017_4896_MOESM1_ESM.docx]

**Optimal visual-haptic integration with articulated tools.**

Chie Takahashi^1^ and Simon J. Watt^2^

^1^ School of Computer Science, University of Birmingham, Birmingham, UK

^2^ Wolfson Centre for Cognitive Neuroscience, School of Psychology, Bangor University, Bangor, UK

**OVERVIEW**

In designing and interpreting the main experiment, we assumed that the degree of conflict between visual and haptic sizes *per se* determines the degree of integration (due to inferring the probability that the signals share a common cause). The main experiment did not test this assumption outwith tool use, and so it warrants validation. Moreover, there was variability across subjects in the reduction in integration caused by visual-haptic conflict. We assumed that this reflected a common, general mechanism across all participants, which considered similarity in visual and haptic sizes, but with different individual ‘tolerances’ to conflict. It could, however, reflect the adoption of idiosyncratic strategies in response to tool use, which would conflict with our interpretation.

To explore this we ran a small-scale control experiment in which we measured the degree of sensory integration as a function of visual-haptic conflict *per se*, without a tool, for three subjects who showed different-sized effects of conflict in the main experiment. When using the tool, visual-haptic conflict (by a factor of 1.6:1) caused subject SE’s performance to change from optimal-integration to no-integration (single-cue) level. Conflict had a smaller effect, however, for subject JB, and in particular subject YY, who showed near-optimal integration in all conditions. We reasoned that if our tool-use data reflect an underlying process that considers visual-haptic size conflict *per se,* we should see the same pattern of differences across individuals whether or not a tool is used.

**METHOD**

Aside from the absence of tools, the procedure for the control experiment was identical to the main experiment, and so we describe only the differences here. Subjects YY, SE, and JB from the main experiment took part. We measured the degree of integration (JNDs in size) as a function of visual-haptic conflict. Haptic size was always 50 mm, and visual sizes were 50, 60, 80, and 100 mm, corresponding to conflicts (visual size/haptic size) of a factor of 1.0, 1.2, 1.6 (same as Experiment 1) and 2.0. We adjusted visual noise values as in the main experiment to match the precision of visual and haptic size estimates.

**RESULTS AND DISCUSSION**

The top row in Figure 6 shows size-discrimination performance for each subject as a function of visual-haptic conflict. For all three subjects, increasing conflict lead to systematic reductions in integration (larger JNDs), but the functions clearly differed. The degree of integration reduced most rapidly with conflict (initially, at least) for subject SE, reducing more slowly for JB, and only a small amount for YY, even at the largest conflict. This is similar to the pattern of effects observed for these subjects in the main experiment. The bottom row in Figure 6 directly compares the effects of the same conflict magnitude (a factor of 1.6) across the two experiments. It can be seen that, within each subject, the same conflict resulted in quantitatively similar reductions in integration, whether or not a tool was used. That is, the difference in individual subject’s ‘tolerances’ to conflict remained without a tool (SE’s integration performance was again highly sensitive to conflict, whereas YY’s was not).

These data confirm that visual-haptic conflicts *per se* do cause a reduction in integration (a premise of our experiment design). Moreover, the similarity of individual subject’s ‘tolerances’ to conflict across tool and no-tool conditions is consistent with a common process in the two cases. In particular, it is notable that YY was similarly ‘insensitive’ to conflict with and without a tool, suggesting his (atypical) pattern of results in the main experiment was not specific to tool use, but reflected a general lack of sensitivity to visual-haptic conflict*.* Overall, these results suggest our tool-use data reflect the operation of a general integration mechanism that considers the similarity of visual and haptic size estimates, and not idiosyncratic strategies specific to tool use.

|  |
| --- |
| **Fig 6** Effect of visual-haptic conflict on discrimination performance in the control experiment (without a tool). The top row shows visual-haptic size JNDs for the three subjects as a function of the conflict ratio (visual size/haptic size). The horizontal solid grey line denotes the observed single-modality JND for each subject, and the dashed grey line denotes the predicted JND if statistically optimal integration occurred (Equation 1 in the main paper). The vertical dashed (red) line illustrates the visual-haptic conflict presented in conditions 2 and 4 of the main experiment. We assumed that conflicts of 80/50 mm (condition 2) and 50/80 mm (condition 4) are equivalent. The solid black curves are the best fitting second-order polynomial function in each case. The bottom row compares the effect of visual-haptic conflict on each subject’s size JNDs across the two experiments. In each plot the left three bars show the data in no-conflict conditions, and the right three bars show the data with a conflict ratio of 1.6. Within each triplet, the left-most (grey) bar shows performance in the control experiment (no-tool). The middle and right-most bars (blue and red) show the same subject’s data from the main experiment, for equivalent conflict conditions, with the 1:1 tool and 1.6:1 tool, respectively. To aid comparison across different tool-gain conditions, the data are plotted as a proportion of each subject’s single-cue performance in each case (shown by the solid grey line). The dashed grey line denotes predicted optimal integration performance, which in these normalised units is equal to 1.0 (single-cue performance)/√2. Error bars denote ±1 standard error  It is also interesting to note that for two subjects (SE and JB) the largest conflict resulted in multisensory discrimination performance that was worse than single-cue performance. We suggest this likely reflects the 2-IFC task used, rather than decreased precision of the underlying unimodal sensory estimates. In the absence of a single, integrated estimate from each interval, subjects face the problem of making a binary decision—which interval contained the taller stimulus?—about four signals in all (two conflicting visual and haptic pairs). If one sensory modality (e.g. vision) could be selected, and the other discarded, performance would simply always remain at single-cue level. Consider if this is not possible, however. Assuming one signal is selected from each interval, at random, and the two signals compared, judgements will sometimes be made *across* sensory modalities (i.e. comparing the haptic size from the first interval with the visual size in the second, and *vice versa*). In our conflict conditions, the visual size was larger than the haptic size (often by more than the difference between standard and comparison stimulus sizes), and so sometimes the visual stimulus would be judged larger than the haptic stimulus, even though the latter came from the ‘larger’ pair, causing worse-than-single-cue performance. |
